# Whole exome sequencing and homozygosity mapping reveals genetic defects in consanguineous Iranian families with inherited retinal dystrophies

**DOI:** 10.1038/s41598-020-75841-9

**Published:** 2020-11-10

**Authors:** Arash Salmaninejad, Nicola Bedoni, Zeinab Ravesh, Mathieu Quinodoz, Nasser Shoeibi, Majid Mojarrad, Alireza Pasdar, Carlo Rivolta

**Affiliations:** 1 Department of Medical Genetics and Molecular Medicine, Faculty of Medicine, Mashhad University of Medical Sciences, Mashhad, Iran; 2 Medical Genetics Research Centre, Faculty of Medicine, Mashhad University of Medical Sciences, Mashhad, Iran; 3 Division of Genetic Medicine, University Hospital of Lausanne, Lausanne, Switzerland; 4 Department of Genetics and Genome Biology, University of Leicester, Leicester, UK; 5 Division of Applied Medicine, Medical School, University of Aberdeen, Foresterhill, Aberdeen, UK; 6 Institute of Molecular and Clinical Ophthalmology Basel (IOB), Basel, Switzerland; 7 Department of Ophthalmology, University of Basel, Basel, Switzerland; 8 Eye Research Center, Mashhad University of Medical Sciences, Mashhad, Iran

**Keywords:** Medical genetics, Mutation, Genetics, Genome, Genetic variation

## Abstract

Inherited retinal dystrophies (IRDs), displaying pronounced genetic and clinical heterogeneity, comprise of a broad range of diseases characterized by progressive retinal cell death and gradual loss of vision. By the combined use of whole exome sequencing (WES), SNP-array and WES-based homozygosity mapping, as well as directed DNA sequencing (Sanger), we have identified nine pathogenic variants in six genes (*ABCA4*, *RPE65*, *MERTK*, *USH2A*, *SPATA7*, *TULP1*) in 10 consanguineous Iranian families. Six of the nine identified variants were novel, including a putative founder mutation in *ABCA4* (c.3260A>G, p.Glu1087Gly), detected in two families from Northeastern Iran. Our findings provide additional information to the molecular pathology of IRDs in Iran, hopefully contributing to better genetic counselling and patient management in the respective families from this country.

## Introduction

Inherited Retinal Dystrophies (IRDs) are a genetically and clinically heterogeneous group of diseases characterized by the progressive loss of retinal photoreceptor cells. This neurodegenerative process leads in turn to increasing visual deficit, and over the years to very poor vision and blindness^1^. With an incidence of approximately 2.5 in 10,000, IRDs are among the most frequently observed hereditary eye diseases^2–4^. Due to their complex genetic and clinical nature, IRDs can be divided into a wide range of clinical subtypes, including: Leber congenital amaurosis (LCA; OMIM #204000), cone-rod dystrophy (CORD; #120970), Stargardt disease (STGD; #248200), retinitis pigmentosa (RP; #268000) and others. They can also manifest as individual eye disorders or involve other organs, as part of various syndromes^5^.

Thus far, mutations in over 270 genes have been associated with IRDs (RetNet. https://sph.uth.edu/retnet/). Recent advances in massively parallel next-generation sequencing (NGS) and genetic testing approaches have substantially improved the identification of mutations, as well as the diagnostic yield of such a genetically heterogeneous condition^6^. Given the intricate nature of IRDs, molecular testing in clinical settings may lead to a more accurate diagnosis of patients with ambiguous ophthalmologic evaluations^7^.

Consanguineous marriages have been globally practiced as a social norm for thousands of years, leading to a high degree of inbreeding in some populations, including Iranians^8^. As a consequence of inter-family marriages, autozygous regions are created through the inheritance of identical-by-descent haplotypes, resulting in turn in a very high prevalence of recessive conditions^9^.

This study aimed at integrating NGS and homozygosity mapping, a technique that identifies long stretches of homozygous haplotypes, for the genetic characterization of IRDs in 10 consanguineous Iranian families.

## Results

**Figure 1:**
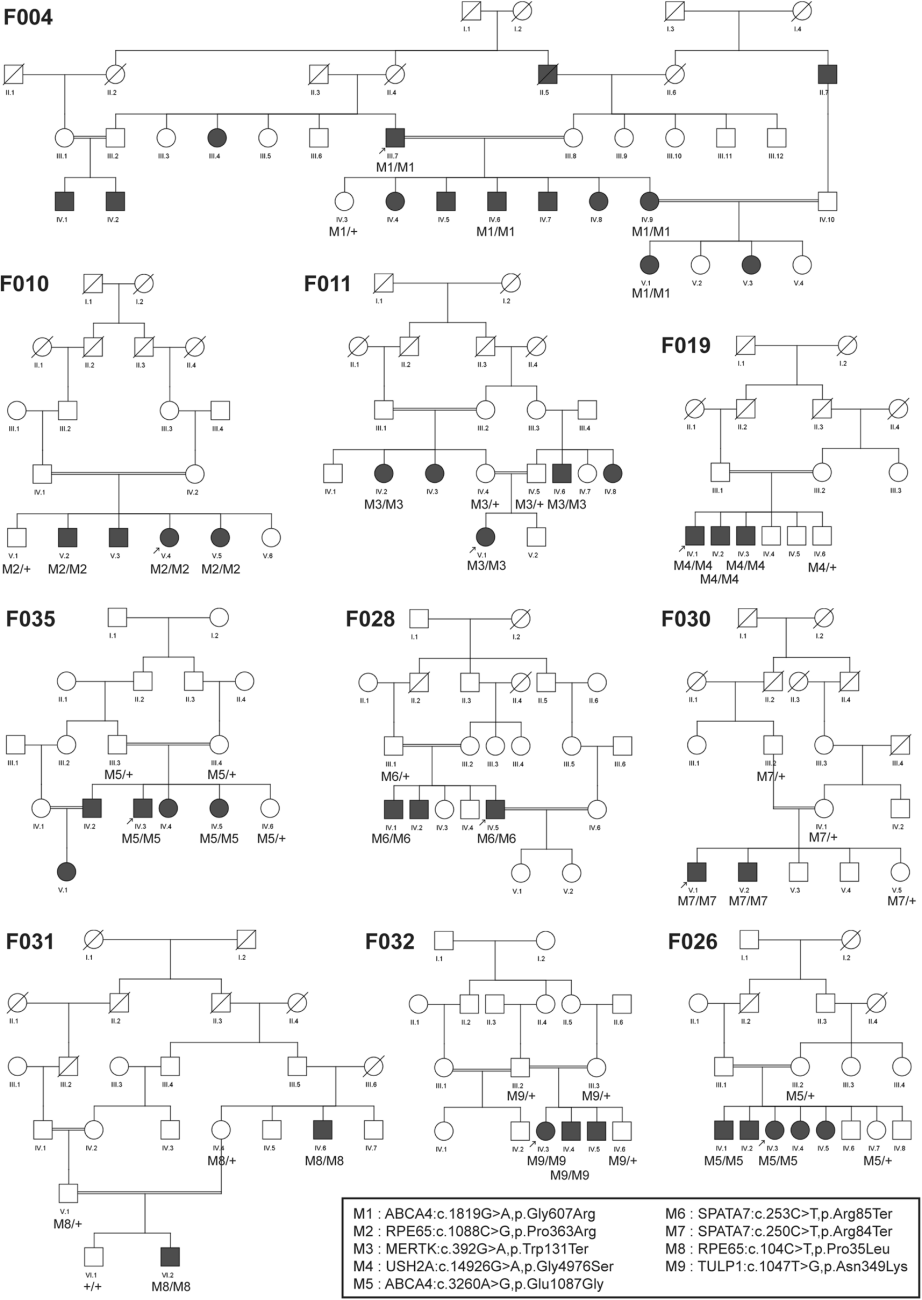
Overview of the pedigrees of the 10 families presented in this study and segregation analysis of the pathogenic variants identified. Probands who underwent WES analysis are indicated with an arrow. Genotypes are also indicated, whenever available.

**Figure 2:**
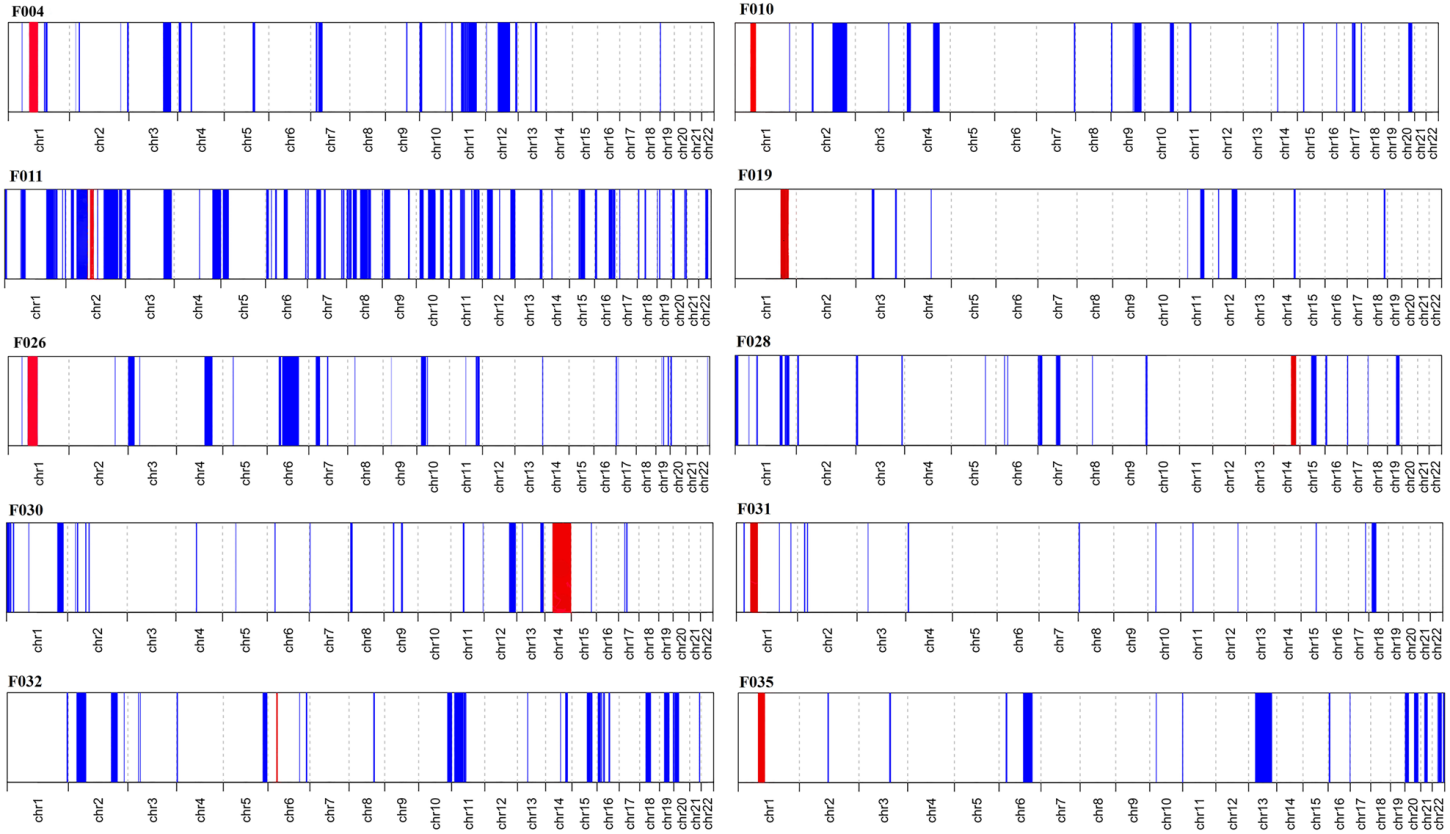
Homozygosity mapping for the 10 probands of this study, generated using the AutoMap tool (Quinodoz et al., manuscript under review). Autozygous regions for autosomes are depicted in blue. Intervals containing the identified pathogenic variants are highlighted in red.

Following WES analysis in the probands from the 10 families ascertained in this study (Fig. 1), we identified nine pathogenic variants in six IRD-associated genes (*ABCA4*, *MERTK*, *RPE65*, *SPATA7*, *TULP1*, and *USH2A*). Of these variants, six were novel, and none of them was previously reported in the Iranian population (Table 1). All of the identified variants were homozygous and resided in autozygous regions of a genomic size of at least 6 Mb, hence reflecting recent endogamy in the population (Table 1, Fig. 2). Autozygome analysis using data from SNP arrays and WES revealed a relatively high number of runs of homozygosity (ROH) (Fig. 2), consistent with the high degree of consanguinity observed in these families (The ROH details for each family containing RetNet genes has been provided in Supplementary Table 1). Autozygome analysis and variant filtering on the WES data was sufficient to identify putative causative variants. SNP data confirmed that the candidate variant located to a homozygous interval in another affected family member. Additional (homozygous) candidate variants filtered for frequency, impact on protein and known to be causative of relevant diseases have been shown in Supplementary Table 2. All findings were validated by Sanger sequencing and, when DNA was available, variant segregation was confirmed within healthy and affected family members, as illustrated in Fig. 1.

**Table 1:** Pathogenic variants identified by WES in probands from 10 Iranian consanguineous families with AR IRD.

Family ID	Mutation number	Gene	RefSeq	Exon	Transcript variant	Protein variant	Zygosity	ROH size [Mb]	Method	References
F004	M1	*ABCA4*	NM_000350.3	13	c.1819G>A	p.Gly607Arg	Hom	35.0	WES + SNP array	^6,10–12^
F010	M2	*RPE65*	NM_000329.3	10	c.1088C>G	p.Pro363Arg	Hom	22.2	WES + SNP array	Novel
F011	M3	*MERTK*	NM_006343.3	2	c.392G>A	p.Trp131Ter	Hom	7.2	WES + SNP array	Novel
F019	M4	*USH2A*	NM_206933.3	68	c.14926G>A	p.Gly4976Ser	Hom	10.9	WES + SNP array	Novel
F026	M5	*ABCA4*	NM_000350.3	22	c.3260A>G	p.Glu1087Gly	Hom	41.2	WES	Novel
F035								27.3	WES	
F028	M6	*SPATA7*	NM_018418.5	5	c.253C>T	p.Arg85Ter	Hom	20.7	WES	^13,14^
F030	M7	*SPATA7*	NM_018418.5	5	c.250C>T	p.Gln84Ter	Hom	74.7	WES + SNP array	Novel
F031	M8	*RPE65*	NM_000329.3	3	c.104C>T	p.Pro35Leu	Hom	30.4	WES + 2SNP array	Novel
F032	M9	*TULP1*	NM_003322.6	11	c.1047T>G	p.Asn349Lys	Hom	6.1	WES + SNP array	ClinVar
										(SCV001161320.1)

In proband V.4 of family F010 (Fig. 1) we identified a novel homozygous missense variant in the *RPE65* gene (NM_000329.3:c.1088C>G, p.Pro363Arg). In this same gene, another novel missense variant was found in proband IV.6 of family F031 (NM_000329.3:c.104C>T, p.Pro35Leu). Both variants are predicted to be pathogenic or likely pathogenic by different prediction tools (Table 2). In family F011, patient V.1 resulted in harboring a homozygous premature stop codon in the *MERTK* gene (NM_006343.3:c.392G>A, p.Trp131Ter), never published before. Family F019 was found to carry a homozygous missense variant in the *USH2A* gene (NM_206933.3:c.14926G>A, p.Gly4976Ser), affecting an evolutionarily conserved amino acid. Similarly, this novel variant was predicted to be pathogenic or likely pathogenic by all tested tools (Table 2).

**Table 2:** Prediction of pathogenicity for all novel variants.

Gene	DNA change	Protein change	Mutation taster	Poly Phen	SIFT	Varsome	CADD score	PhyloP score	Provean	Allele frequency in databases			
										Iranome (N = 800)	EVS (N = 6515)	gnomAD(N = 141,456)	1,000Genomes
*ABCA4*	c.3260A>G	p.Glu1087Gly	DC	PD	Dam	P	32	6.22	Del	Not found	Not found	0.00003	Not found
*RPE65*	c.1088C>G	p.Pro363Arg	DC	PD	Dam	P	28.0	5.76	Del	Not found	Not found	Not found	Not found
	c.104 C>T	p.Pro35Leu	DC	PD	Dam	LP	30	5.77	Del	Not found	Not found	Not found	Not found
*SPATA7*	c.250 C>T	p.Gln84Ter	DC	NA	NA	P	37	2.51	NA	Not found	Not found	Not found	Not found
*MERTK*	c.392G>A	p.Trp131Ter	DC	NA	NA	P	37	3.01	NA	Not found	Not found	0.000003	Not Found
*USH2A*	c.14926G>A	p.Gly4976Ser	DC	PD	Dam	LP	25.4	5.8	Del	Not found	Not found	0.00008	Not Found

*Dam* damaging; *DC* disease causing; *Del* deleterious; *LP* likely pathogenic; *P* pathogenic; *PD* probably damaging; *NA* not applicable.

Our NGS analyses revealed pathogenic variants in the LCA-associated genes *SPATA7* and *TULP1*. Proband IV.5 of family F028 had a homozygous premature stop codon in *SPATA7* (NM_018418.5:c.253C>T, p.Arg85Ter), previously described to cause LCA in three consanguineous families of Pakistani and Bangladeshi origins^13^. Segregation was confirmed in the other affected sibling and in the healthy father, who was heterozygous. Similarly, affected members of family F030 carried another homozygous premature stop codon in the same gene (NM_018418.5:c.250C>T, p.Gln84Ter). Sanger sequencing with available DNA samples confirmed that the parents and the healthy sister were both heterozygous carriers (Fig. 1). Moreover, in proband IV.3 of family F032 we identified a known pathogenic missense variant in the *TULP1* gene (NM_003322.6:c.1047T>G, p.Asn349Lys). Again, segregation analysis was consistent with the recessive mode of inheritance of this disease (Fig. 1).

WES analysis of patient III.7 of family F004 identified a homozygous missense variant (NM_000350.3:c.1819G>A, p.Gly607Arg) in exon 13 of the *ABCA4* gene. This same variant was previously found in patients with STGD^10,15^. Segregation was confirmed, as shown in Fig. 1. Lastly, in families F026 and F035 we identified a previously-unreported missense variant (NM_000350.3:c.3260A>G, p.Glu1087Gly) in exon 22 of the *ABCA4* gene. Interestingly, this same codon was found to carry two missense variants in patients with this condition in previous publications^10,16–19^ as well as a nonsense variant in patients with cone-rod dystrophy^20^.

## Discussion

Consanguineous marriages have been traditionally practiced in Iran as a consequence of socio-cultural factors. It is estimated that nearly 40% of Iranian marriages are between related individuals, of which ~ 21% are first cousins and ~ 19% are second cousins^21,22^. Endogamy, consanguinity or geographic isolation may rise the occurrence of specific mutations in selected populations, which can be isolated by homozygosity mapping. The rationale for this method is that unaffected parents who have some degree of kinship, belong to an ethnic group with high endogamy or are from a geographical isolate, could be heterozygotes for the same recessive mutation from a common ancestor. This mutation, which at the population level possibly will even be infrequent, can be brought to homozygosity since consanguinity can cause disease in these parents’ children^23,24^. This feature makes the Iranian gene pool one of the richest resources for genetic investigations. Several studies have so far been published concerning the genetic basis of IRDs in Iran^25–30^. In this study, we further refined the genetic landscape of IRDs by identifying nine pathogenic variants in genes that were previously associated with these conditions.

In particular, we identified a putative novel founder missense mutation (p.Glu1087Gly) in the *ABCA4* gene, within individuals from the Northeastern region (Khorasan province) of Iran. They had a similar ethnic origin and shared an identical homozygous haplotype around *ABCA4* (chr1:85′647′942–97′544′759 of size 11.9 Mb). The phenotypic features of the probands and their affected relatives were associated with those of classical STGD. According to data extracted from the database of protein families (Pfam), amino acids 946–1090 encode the ABC Transporter wherein based on the arrangement of the Pfam domains, the 1087 position is conserved and predicted to be an active site (https://pfam.xfam.org/protein/ABCA4_HUMAN). Two missenses affecting the same amino acid were already found to be pathogenic: p.Glu1087Asp and p.Glu1087Lys^31,32^. In addition to the increasing number of reports on pronounced clinical and genetic variability of genes that cause IRDs, mutations in three different genes, *RPE65*, *MERTK* and *USH2A* were ascertained in four unrelated families with members showing symptoms of RP. Fortunately, recent advances in gene therapy trials on patients with *RPE65* and *MERTK* mutations have shown promising results, reflecting hope for a potentially revolutionary form of therapy in a subset of patients^33–36^. Our study stipulates the role of these variants in the pathogenesis of RP in Iranian patients, who could potentially be candidates for future therapeutic interventions. Furthermore, genetic alterations in *SPATA7* and *TULP1* are known to manifest with overlapping clinical symptoms of LCA and RP^37,38^. Evaluating the current status of detected variants and also checking them against other Iranian databases which showed nil frequencies provides supporting evidence that these variants can be classed as potential causative variants.

The advent of gene-directed interventions has enabled bridging of molecular genetics with genetic counseling and possible therapeutic interventions in ophthalmology. Specifically, since targeted gene therapy is constantly advancing, detection of genetic mutations may soon be a routine procedure to allow patients to access suitable therapies.

In this context, our study expands the current understanding of the molecular basis of IRDs in Iran, while providing at the same time elements of information for genetic and prenatal counselling as well as possible indications for future therapy.

## Materials and methods

### Ethics statement

This study was designed in compliance with the tenets of the Declaration of Helsinki. Written informed consent was obtained from each participant or legal guardian prior to their participation. This study was approved by the Institutional Review Boards of our respective Universities: Mashhad University of Medical Sciences (MUMS), and University of Lausanne.

### Families and preparation of samples

Families were selected according to three main criteria: (i) they included two or more individuals with confirmed IRD diagnosis, (ii) showed a likely autosomal recessive (AR) pattern of inheritance, and (iii) had a history of consanguinity. Pedigrees were drawn using the Pedigree Chart Designer software (CeGaT, Tubingen, Germany). Clinical information and clinical data were extracted from patients’ medical records of Mashhad Khatamolanbia Eye Hospital. All patients were evaluated by an ophthalmologist and Optical Coherence Tomography (OCT) was performed for individuals who had no recent follow up. Six milliliters of peripheral blood from available affected and unaffected members of each family were collected and mixed with EDTA (Merck KGaA, Darmstadt, Germany). A phenol–chloroform method was used to extract genomic DNA from peripheral leukocytes. DNA quality and quantity were verified using a Nanodrop 2000 (Thermo Fisher Scientific, Wilmington, DE, USA). DNA integrity was ascertained by running DNAs on 1% agarose gel. Samples were stored at − 20 °C until used.

### SNP genotyping

DNAs of studied individuals were genotyped at the iGE3 Platform of the University of Geneva, Switzerland, using an Illumina Infinium array (San Diego, CA, USA; GSAMD-24v2.0). Genotype values were obtained with GenomeStudio (Illumina). Homozygosity mapping was obtained by the use of the PLINK software. Due to the high degree of consanguinity, SNP genotyping was chosen as a complementary analysis to narrow down homozygous regions and reduce the number of candidate variants.

### Whole-exome sequencing

WES was performed on one designated proband from each of the families analyzed. Exome capture and library preparation was performed using the SureSelect Human All Exon v6 kit (Agilent, Santa Clara, CA, USA) and the HiSeq Rapid PE Cluster Kit v2 (Illumina, San Diego, CA, USA), from 2 μg genomic DNA. Libraries were sequenced on a HiSeq 2500 instruments (Illumina). Raw reads were mapped to the human genome reference sequence (build hg19) with the Novoalign software (V3.08.00, Novocraft Technologies, Selangor, Malaysia). Duplicate reads were then removed using Picard (v. 2.14.0-SNAPSHOT). Base quality score recalibration was performed with HaplotypeCaller (GATK, v.4.0.3.0). Single nucleotide variants (SNVs) and small insertions and deletions were detected using the Genome Analysis Tool Kit (GATK v4.0) software package, using the Best Practice Guidelines identified by the developers^39^. ExomeDepth software was used to detect exonic deletions. Homozygous variants in IRD genes (according to RetNet) lying within ROHs were processed as follows. Filtration was performed based on quality (DP > 10, GQ > 30, FS < 25 and alternative read percentage > 25%) and allelic frequency [below 1% in ExAC, gnomAD, 1000 Genomes, ESP (NHLBI Exome Variant Server, https://evs.gs.washington.edu/EVS), GME (GME Variome https://igm.ucsd.edu/gme/index.php), and ABraOM]. The pathogenicity of the genetic variants was assessed after functional annotation through ANNOVAR^40^ and using in-house scripts^41^, where the predicted impact on protein sequence and messenger RNA (mRNA) splicing were also taken into account. Finally, we checked the frequency of variants in the Iranian population using the Iranome database (https://www.iranome.ir/). ROHs were identified by using the AutoMap software (Quinodoz et al., manuscript under review. https://automap.iob.ch/) on WES data. The number of filtered variants at each step is shown in Supplementary Table 3.

### Sanger validation and segregation analysis

Primers were designed using the Primer3 online software^42^, at least 70 bp upstream and downstream of the variants, and PCR reactions were performed under standard conditions (for the list of primers see Supplementary Table 4). PCR products were purified by treatment with exonuclease and thermosensitive alkaline phosphatase (Thermo Fisher Scientific, Waltham, MA, USA) and analyzed by Sanger sequencing using the Big Dye Terminator v3.1 Cycling Sequencing Kit (Applied Biosystem, Foster City, CA, USA) on an ABI 3730XL platform (Applied Biosystems). Sequencing data were analyzed using the Sequencher v4.8, SnapGene (https://www.snapgene.com), and Chromas Lite v2.01 software and compared with wild-type samples and reference sequences from NCBI and Ensembl databases. Furthermore, frequency of all candidate variants was verified in a database of 800 Iranian healthy individuals (https://www.iranome.ir/).

## Supplementary information

Supplementary Table 1.

Supplementary Table 2.

Supplementary Table 3.

Supplementary Table 4.
