# Supplementary material for: Whole exome sequencing and homozygosity mapping reveals genetic defects in consanguineous Iranian families with inherited retinal dystrophies
Source: Sci Rep. 2020 Nov 10;10:19413. doi: 10.1038/s41598-020-75841-9 (PMC7655865; doi:10.1038/s41598-020-75841-9)
Supplement: Supplementary file 3 — Supplementary Table 3. [file 41598_2020_75841_MOESM3_ESM.docx]

**Whole Exome Sequencing and Homozygosity Mapping Reveals Genetic Defects in Consanguineous Iranian Families with Inherited Retinal Dystrophies**

Arash Salmaninejad^1,2 §^, Nicola Bedoni^3 §^, Zeinab Ravesh^4^, Mathieu Quinodoz^4,6,7^, Nasser Shoeibi^8^, Majid Mojarrad^1,2^, Alireza Pasdar^1,2,5 *^, Carlo Rivolta^4,6,7 *^

1 Department of Medical Genetics and Molecular Medicine, Faculty of Medicine, Mashhad University of Medical Sciences, Mashhad, Iran.

2 Medical Genetics Research Centre, Faculty of Medicine, Mashhad University of Medical Sciences, Mashhad, Iran.

3 Division of Genetic Medicine, University Hospital of Lausanne, Lausanne, Switzerland

4 Department of Genetics and Genome Biology, University of Leicester, Leicester, United Kingdom.

5 Division of Applied Medicine, Medical School, University of Aberdeen, Foresterhill, Aberdeen, United Kingdom.

6 Institute of Molecular and Clinical Ophthalmology Basel (IOB), Basel, Switzerland.

7 Department of Ophthalmology, University of Basel, Basel, Switzerland.

8 Eye Research Center, Mashhad University of Medical Sciences, Mashhad, Iran.

^§^ Equal contribution

*Correspondence:

Alireza Pasdar

Department of Medical Genetics, Faculty of Medicine, Mashhad University of Medical Sciences, Mashhad, Iran.

E-mail address: [pasdara@mums.ac.ir](mailto:pasdara@mums.ac.ir); [a.pasdar@abdn.ac.uk](mailto:a.pasdar@abdn.ac.uk) Tel / Fax: +985138002310

Carlo Rivolta

Institute of Molecular and Clinical Ophthalmology Basel (IOB), Basel, Switzerland

E-mail address: carlo.rivolta@iob.ch Tel. +41 43 215 2795

**Supplementary Table 3.** Filtration stages and number of variants at each step

| Filtration  Family | Raw number of variants | MAF < 1% | High quality | Impact at the protein level | Homozygous | RetNet | IRD / Recessive / Segregation |
| --- | --- | --- | --- | --- | --- | --- | --- |
| F004 | 82314 | 2456 | 1085 | 392 | 19 | 2 | 1 |
| F010 | 91673 | 2546 | 1227 | 399 | 21 | 1 | 1 |
| F011 | 85720 | 2428 | 1042 | 331 | 55 | 1 | 1 |
| F019 | 87305 | 2546 | 1198 | 434 | 7 | 1 | 1 |
| F026 | 92990 | 2443 | 1181 | 407 | 20 | 2 | 1 |
| F028 | 82319 | 2618 | 1122 | 433 | 18 | 1 | 1 |
| F030 | 92828 | 2550 | 1255 | 418 | 22 | 1 | 1 |
| F031 | 89633 | 2646 | 1154 | 411 | 6 | 1 | 1 |
| F032 | 85760 | 2175 | 1006 | 356 | 28 | 3 | 1 |
| F035 | 79344 | 2333 | 943 | 350 | 10 | 1 | 1 |

MAF: Minor Allele Frequency; ROH: Run Of Homozygosity; IRD Inherited Retinal Degenerations
