# Supplementary material for: Whole exome sequencing and homozygosity mapping reveals genetic defects in consanguineous Iranian families with inherited retinal dystrophies
Source: Sci Rep. 2020 Nov 10;10:19413. doi: 10.1038/s41598-020-75841-9 (PMC7655865; doi:10.1038/s41598-020-75841-9)
Supplement: Supplementary file 4 — Supplementary Table 4. [file 41598_2020_75841_MOESM4_ESM.docx]

**Whole Exome Sequencing and Homozygosity Mapping Reveals Genetic Defects in Consanguineous Iranian Families with Inherited Retinal Dystrophies**

Arash Salmaninejad^1,2 §^, Nicola Bedoni^3 §^, Zeinab Ravesh^4^, Mathieu Quinodoz^4,6,7^, Nasser Shoeibi^8^, Majid Mojarrad^1,2^, Alireza Pasdar^1,2,5 *^, Carlo Rivolta^4,6,7 *^

1 Department of Medical Genetics and Molecular Medicine, Faculty of Medicine, Mashhad University of Medical Sciences, Mashhad, Iran.

2 Medical Genetics Research Centre, Faculty of Medicine, Mashhad University of Medical Sciences, Mashhad, Iran.

3 Division of Genetic Medicine, University Hospital of Lausanne, Lausanne, Switzerland

4 Department of Genetics and Genome Biology, University of Leicester, Leicester, United Kingdom.

5 Division of Applied Medicine, Medical School, University of Aberdeen, Foresterhill, Aberdeen, United Kingdom.

6 Institute of Molecular and Clinical Ophthalmology Basel (IOB), Basel, Switzerland.

7 Department of Ophthalmology, University of Basel, Basel, Switzerland.

8 Eye Research Center, Mashhad University of Medical Sciences, Mashhad, Iran.

^§^ Equal contribution

*Correspondence:

Alireza Pasdar

Department of Medical Genetics, Faculty of Medicine, Mashhad University of Medical Sciences, Mashhad, Iran.

E-mail address: [pasdara@mums.ac.ir](mailto:pasdara@mums.ac.ir); [a.pasdar@abdn.ac.uk](mailto:a.pasdar@abdn.ac.uk) Tel / Fax: +985138002310

Carlo Rivolta

Institute of Molecular and Clinical Ophthalmology Basel (IOB), Basel, Switzerland

E-mail address: carlo.rivolta@iob.ch Tel. +41 43 215 2795

**Supplementary Table 4.** List of primer sequences used for PCR and Sanger sequencing (segregation analysis). Annealing temperature for PCRs was 59 C, for all amplicons.

| Gene | Exon | Variant | Direction | Primer sequence (5’-3’) | Amplicon Size (bp) |
| --- | --- | --- | --- | --- | --- |
| *ABCA4* | Exon 13 | p.Gly607Arg | F | CCCATCCTTTGTCCCTCTGT | 247 |
|  |  |  | R | CCTCCTCCATGACACGCTAA |  |
|  | Exon 22 | p.Glu1087Gly | F | GCCTTTCTCTTCCTCACCCT | 277 |
|  |  |  | R | AATGGCAGGTGAGAGAGTGG |  |
| *RPE65* | Exon 10 | p.Pro363Arg | F | ATGGCTCTGATACACCTGGC | 352 |
|  |  |  | R | ACATGAGGCAGGAGGACAAT |  |
|  | Exon 3 | p.Pro35Leu | F | CACTGCCAGCTCTATGAGGA | 241 |
|  |  |  | R | GTCAAACTTGTGCAGGAGGG |  |
| *MERTK* | Exon 2 | p.Trp131Ter | F | TCTGTCGAATCAAAGCCCCT | 380 |
|  |  |  | R | CGTTTGAACTCAGGAGGTGG |  |
| *USH2A* | Exon 68 | p.Gly4976Ser | F | CTGTGGTGTGTGTGAACTGG | 322 |
|  |  |  | R | TCAAGCTGCAAATGGGAAGG |  |
| *SPATA7* | Exon 5 | p.Gln84Ter | F | TGGCCAGTTTGTCATATCTTGTT | 656 |
|  |  | p.Arg85Ter | R | ACTGTCTATGTCTGCCTTCCA |  |
| *TULP1* | Exon 11 | p.Asn349Lys | F | ACCTGGACACGGAGAAGAAG | 349 |
|  |  |  | R | ACAGAGATGACGGGCTATGG |  |

F: Forward, R: Reverse
